# Preparation and Characterization of Oxide Coatings with LDH Nanosheets on AZ91 Magnesium Alloy by a One-Step Low Voltage Microarc Oxidation Process

**DOI:** 10.3390/ma19020216

**Published:** 2026-01-06

**Authors:** Longfeng Shi, Xuchen Lu, Peixuan Li, Cancan Liu, Jun Liang

**Affiliations:** 1 College of Materials Science and Engineering, Nanjing Tech University, Nanjing 211816, China; 17706217736@163.com (L.S.); xuchenlu113@163.com (X.L.); 2 Research Institute of Interdisciplinary Science, School of Materials Science and Engineering, Dongguan University of Technology, Dongguan 523808, China; 17835617674@163.com

**Keywords:** low voltage microarc oxidation, magnesium alloy, LDH nanosheets, corrosion resistance

## Abstract

In this study, oxide coatings with layered double hydroxide (LDH) nanosheets were prepared on AZ91 magnesium alloy by a one-step low-voltage microarc oxidation (MAO) process. The microstructure and composition of the coatings were characterized using SEM, EDS, XRD, FT-IR, and XPS. The corrosion protection performance of the coatings was evaluated by electrochemical analysis and hydrogen evolution tests. The results showed that oxide coatings with Mg-Al-LDH nanosheets are successfully produced by microarc oxidation at a voltage of less than 100 V. The coating with a higher density of Mg-Al LDH nanosheets exhibited enhanced corrosion resistance. Moreover, after modification with stearic acid, the coatings displayed high hydrophobicity and corrosion resistance.

## 1. Introduction

Magnesium alloy is recognized as an ideal lightweight material owing to its high specific strength, low density, good thermal conductivity, and excellent damping capacity. It is extensively utilized in aerospace, electronic equipment, and vehicles [1,2,3,4,5,6]. However, magnesium has a relatively low standard electrode potential (−2.372 V). Furthermore, the naturally formed oxide layer on its surface is not effectively protective, thereby significantly limiting its widespread application. Consequently, enhancing the corrosion resistance of magnesium alloys has emerged as a critical research focus in materials science.

Surface treatment represents a crucial technique for enhancing the corrosion resistance of magnesium alloys [7,8]. Other such techniques include anodizing [9], chemical conversion [10,11], vapor phase deposition [12], alkaline passivation [13,14], thermal spraying [15], laser surface treatment [16], microarc oxidation [17,18,19,20,21,22,23,24], and so on. Among them, microarc oxidation (MAO) has attracted wide attention for its simple process and high efficiency. The instantaneous high temperature and pressure generated by plasma discharge trigger a series of chemical reactions on the substrate, leading to the in situ formation of an oxide ceramic coating [25,26,27,28,29]. However, the discharge during MAO process often results in various defects, including micropores and cracks. Under service conditions, corrosive agents (e.g., Cl^−^ ions) permeate through coating defects such as micropores and microcracks, initiating localized corrosion of the underlying Mg alloy substrate. Therefore, strategies to enhance the protective performance of MAO coatings should be explored to improve the corrosion resistance of Mg alloys.

To further improve corrosion protection, layered double hydroxides (LDHs) have been applied as a top layer on MAO-coated Mg alloys due to their anion-exchange capacity and barrier properties. An LDH consists of a positively charged brucite-like host layer and a negatively charged anion with a chemical formula of [$M_{2+}$M3x+x1−(OH)_2_][A^n−^] x/*n*·zH_2_O [30,31,32,33,34,35]. LDHs are widely employed in the disciplines of catalysts, adsorbents, and supercapacitors because of their simple chemical composition and strong anion exchange capacity [36,37,38]. An LDH film applied to the MAO coating surface on Mg alloy can effectively seal defects, including micropores and fractures, either completely or partially [34,39,40]. Moreover, its unique ion-exchange property allows it to wrap anions in the corrosive solution, thereby effectively reducing the corrosion of the magnesium alloy.

Hydrothermal treatment is a common method for preparing LDH coatings on MAO coatings [41,42]. For instance, Wang et al. successfully fabricated Mg-Al LDH/MAO composite coatings in a NaNO_3_ solution using the hydrothermal method, which effectively enhanced the corrosion resistance of the coating [43]. Jiang et al. prepared a molybdate-intercalated Mg-Al LDH film on the MAO surface via a hydrothermal process, which demonstrated a significant sealing effect on the porous defects of the MAO coating and served as a carrier for inhibitor loading to achieve active protection [44]. However, the hydrothermal method generally requires high temperature and pressure conditions, often exceeding 120 °C and 1.21 × 10^5^ Pa, which may cause issues such as cracking, spalling, or degradation of the MAO coating. Currently, the production of MAO-LDH coatings on magnesium alloys often involves multiple steps and is limited by high-temperature preparation conditions. During the hydrothermal process, uneven temperature distribution, non-uniform solution flow, or inconsistent reactant distribution may lead to the formation of non-uniform LDH films, thereby affecting their overall quality. Recently, Zhang et al. fabricated MAO/Mg-Al LDH composite coatings using a one-step MAO process by adjusting the electrolyte concentration (Na_3_PO_4_∙12H_2_O) [45]. This method avoided issues related to coating uniformity, but high-voltage MAO is associated with high overall energy consumption. Therefore, further research is needed to develop new methods with lower processing requirements.

In this study, a novel one-step MAO method was developed to grow LDH nanosheets in situ on magnesium alloys. By controlling the electrolyte concentration under ultra-low voltage conditions (less than 100 V), the fabrication of LDH nanosheets on the MAO coating was successfully achieved. Electrochemical characterization (potentiodynamic polarization/EIS) coupled with hydrogen evolution measurements demonstrated that the increased density of LDH nanosheets within the coating substantially improved the corrosion resistance of AZ91 Mg alloy. This enhancement is attributed to the nanosheets’ barrier effect against aggressive ion penetration and their ability to facilitate protective film formation at the metal–electrolyte interface.

## 2. Experiment

### 2.1. Materials

In this work, AZ91D magnesium alloy, with a nominal composition of Al 8.7 wt.%, Zn 0.82 wt.%, Mn 0.27 wt.%, and Si 0.05 wt.% (impurities: Cu < 0.025 wt.%, Fe < 0.004 wt.%, Ni < 0.001 wt.%, Mg balance), was employed as the substrate material. Rectangular specimens (30 mm × 20 mm × 6 mm) were sectioned for subsequent surface treatments. Prior to coating deposition, all substrates underwent sequential mechanical polishing using SiC abrasive papers with progressively finer grit sizes (600# → 800# → 1200# → 1500#). The polished surfaces were then ultrasonically cleaned in ethanol for 15 min to remove particulate contaminants, followed by air drying at room temperature (25 ± 2 °C) in a laminar flow hood.

### 2.2. MAO Coating Preparation

**Figure 1:**
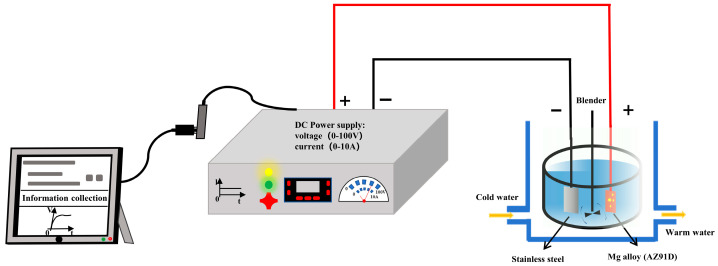
Schematic diagram of the process for preparing the coatings.

A low-voltage DC MAO process was carried out for preparing MAO coating. The preparation process of the coating is schematically illustrated in Figure 1. A two-electrode setup was used, with AZ91 Mg alloy as the anode and stainless steel as the cathode. Constant current mode was used with a current density of 2.78 A/dm^2^, the oxidation time was 8 min, and the water cooler temperature was set to 20 °C. For the MAO process, the electrolyte solution was prepared using 200 g of NaOH per liter of deionized water. In addition, 15 mL, and 30 mL of C_3_H_8_O_3_ were added to the solution as additive. Electrolytic solutions without and with different concentrations of C_3_H_8_O_3_ were designated as MAO–0, MAO–15, and MAO–30, with respective conductivities of 754 mS/cm, 716 mS/cm, and 660 mS/cm.

Surface modification of the MAO coating was performed via an immersion method. using an ethanol-based solution of low-surface-energy substances for surface modification. Specifically, a 100 mL 10 g/L ethanol solution of stearic acid (STA) was prepared first, the MAO samples were vertically immersed in the aforementioned solution for treatment by immersion at 80 °C for 120 min, and the samples obtained were denoted as MAO–0–STA, MAO–15–STA, and MAO–30–STA, respectively.

### 2.3. Characterization

The surface and cross-sectional morphology, along with the chemical composition of the coatings, were analyzed using a field emission scanning electron microscope (Waltham, MA, USA) equipped with an energy-dispersive X-ray spectrometer (Waltham, MA, USA). Additionally, the surface porosity of the coatings was quantified through the standard threshold segmentation method applied to SEM images, with computational analysis conducted using Image Pro Plus software V7.0 (Silver Spring, MD, USA). For cross-sectional observation, the Mg alloy samples were firstly cut and immersed in epoxy resin with the cross-section ground to 1500# with SiC sandpapers, followed by successive polishing with diamond polishing compound to provide a mirror-like surface. The samples were coated with gold to prevent the coating from having poor electrical conductivity prior to testing.

The crystal phases of the coatings were characterized by an X-ray diffractometer (Almelo, Overijssel, The Netherlands) with a Cu target (λ = 0.154 nm) set at an incidence angle of 0.5° and the 2θ range from 5° to 90° with the scanning rate of 5°/min. Both the chemical composition and the chemical state of the coatings were analyzed by X-ray photoelectron spectroscopy (Waltham, Massachusetts, UK). The functional group composition of the coatings was analyzed by Fourier Transform Infrared spectroscopy (Ettlingen, Baden-Württemberg, Germany) in the range of 4000~400 cm^−1^. Meanwhile, the water contact angle (CA) was measured with an optical contact angle meter (Shanghai, China) by depositing a 3 μL droplet onto the sample surface. The reported CA value represents the average of measurements taken at three random locations.

### 2.4. Corrosion Test

The corrosion resistance of the coatings was evaluated in 3.5 wt.% NaCl solution using a three-electrode electrochemical system (JinJiang, TaiZhou Province, China). The electrochemical behavior and protective properties of the coatings were evaluated via potentiodynamic polarization (PDP) and electrochemical impedance spectroscopy (EIS) measurements. The samples were employed as the working electrodes, with an exposure area of about 1 cm^2^, a Pt sheet, and silver/saturated silver chloride (Ag/AgCl) as counter electrodes and reference electrodes, respectively, to perform the electrochemical tests. The Tafel polarization test was performed at a scan rate of 1 mV/s, and the data were recorded from −0.5 V to 0.5 V. The EIS curves were fitted and analyzed with Zview software (3.5.0.10). To evaluate long-term corrosion resistance, hydrogen evolution tests were performed on the samples. Samples were immersed in 3.5 wt.% NaCl solution for hydrogen evolution measurement tests, placed in a beaker, and stored at room temperature, and a gas collection device consisting of a funnel and an acid bucket was used to collect the hydrogen released. Three sets of parallel tests were carried out to ensure repeatability of data.

## 3. Results and Discussion

### 3.1. Characteristics of Low-Voltage MAO Processes

**Figure 2:**
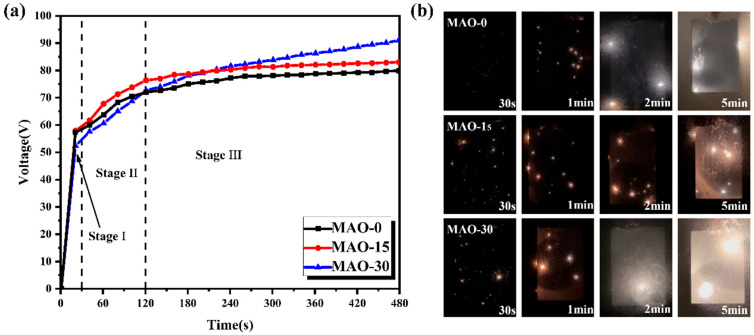
The voltage–time curves (**a**) and corresponding surface discharge characteristics (**b**) of the MAO process on AZ91 Mg alloy in electrolytes with different concentrations of C_3_H_8_O_3_.

The evolution of voltages with time for the low-voltage MAO processes of AZ91 Mg alloy in the electrolytes with the addition of 0 mL, 15 mL, and 30 mL of glycerol (C_3_H_8_O_3_) is depicted in Figure 2. It is clear that the voltage–time curves for these different electrolytes exhibit a similar trend, which can be roughly divided into two stages: a rapid increase stage (stage I) and a gradual increase stage (stage II). During stage I, the voltage rapidly rises to approximately 60 V within 30 s for all MAO processes in the different electrolytes, indicating the formation of passive or barrier layers on the surface of the AZ91 Mg alloy [46]. Subsequently, the voltages gradually increase with the processing time until the end of the process (stage II). However, in stage II, the MAO processes in the electrolytes with the addition of 15 mL and 30 mL C_3_H_8_O_3_ show slightly higher voltages compared to the base electrolyte. The final voltages for the 8 min MAO processes of AZ91 Mg alloy in different electrolytes range from 75 V to 90 V. The surface discharge images of the samples during the MAO processes are shown in Figure 2b. The surface discharge state of the sample tends to stabilize after 5 min, which corresponds to the later stage where the voltage–time curve becomes stable (Figure 2a). For the MAO-0 sample, the discharges on the surface gradually evolve from small, uniform silver-white sparks in the early stage to larger, localized arcs. In comparison, the discharges on the surfaces of MAO–15 and MAO–30 samples are more intense in the early stage, with more yellow arcs appearing in the discharge process. The MAO–15 sample exhibits more uniform discharges in the late stage of MAO process, while the MAO–30 sample shows a localized accumulation of arcs after 2 min of discharge, which continues to increase and lead to localized large arc discharges.

Notably, the arc initiation and final voltages of MAO processes in these electrolytes remain within 90 V, achieving the preparation of MAO coatings at very low voltages. This is primarily attributed to the high conductivity of the solution, resulting from the high concentration of the electrolyte. Meanwhile, the electrolytes with 15 mL and 30 mL of C_3_H_8_O_3_ have higher final voltages than the base electrolyte. Previous studies have shown [47] that the initial voltage of MAO is related to the conductivity of the solution. The decrease in solution conductivity with increasing C_3_H_8_O_3_ content is mainly attributed to the enhanced adsorption of negative ions per unit area at the anode–electrolyte interface. This promotes the formation of dense and uniform discharge centers on the passive film surface [48].

### 3.2. Microstructure and Compositions of Coatings

**Figure 3:**
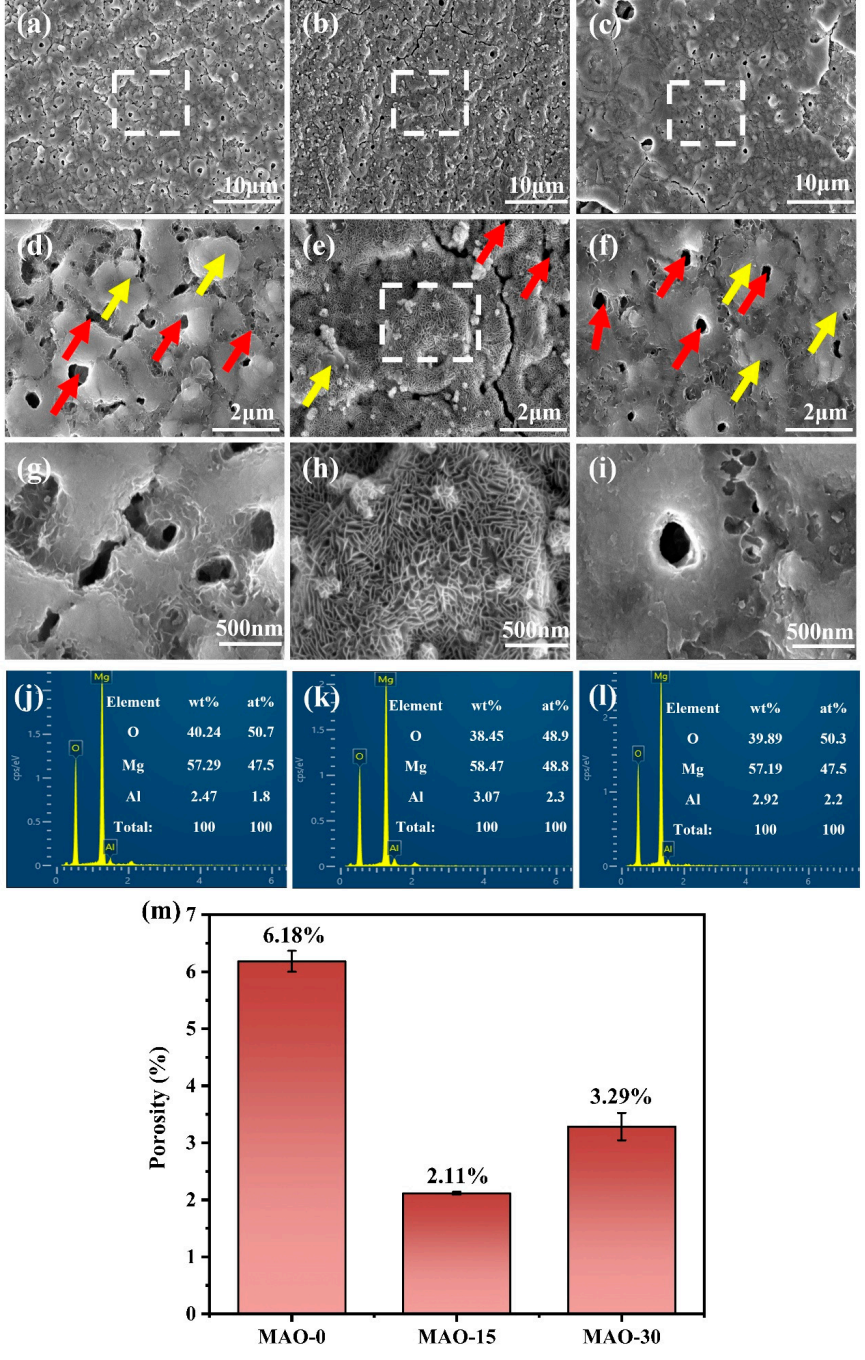
Surface SEM morphologies and corresponding EDS spectra of oxide coatings: (**a**,**d**,**g**,**j**) MAO–0; (**b**,**e**,**h**,**k**) MAO–15; (**c**,**f**,**i**,**l**) MAO–30. (**m**) Surface porosity of the coatings.

The surface morphologies of the MAO coatings are shown in Figure 3. As observed in Figure 3a–c, the coating surface exhibits the presence of pores and cracks. However, the porosity of these coatings is significantly lower than that of conventional microarc oxidation coatings [45]. The micropores formed during the MAO process arise primarily from plasma discharge and the evolution of gas bubbles [49]. Upon local zooming, Figure 3d–f reveal tiny crater-like apertures (marked by red arrows) on the coatings, formed by the ejection of molten oxide during plasma discharge. In addition, the ejected molten oxide is cold-deposited near the pores, forming oxide nodules (shown by yellow arrows), and a few cracks can be observed in the vicinity. While the lamellar surface features remain indistinct at this magnification level, their presence is clearly identifiable in Figure 3e. Subsequent higher-magnification imaging of the framed regions in Figure 3d–f reveals detailed morphological characteristics, as presented in Figure 3g–i.

Figure 3h reveals the presence of aggregated lamellar structure on the surface of the MAO–15 coating, primarily consisting of a large number of nanosheets. These nanosheets grow vertically from the alloy substrate surface of the MAO-coated AZ91. Similar nanosheets are also observed on the surfaces of the MAO–0 and MAO–30 coatings (Figure 3g,i), but their quantity is much smaller compared to that of the MAO–15 coating. Moreover, the nanosheets on the surface of the MAO–30 coating distribute near the cracks and exhibit a tendency to melt. This is because the voltage of microarc oxidation rises as the C_3_H_8_O_3_ increases, resulting in large, localized arc discharges during the MAO process. Under the high-temperature effect of arc discharge, the nanosheets tend to melt on the coating surface. As presented in Figure 3j–l, the surface morphologies and corresponding EDS results of the MAO–0, MAO–15, and MAO–30 coatings reveal that they are primarily composed of Mg and O. In addition, a small amount of Al element can be detected in the coatings, suggesting that Al from the AZ91 magnesium alloy substrate participates in the reaction during the MAO process. Overall, the MAO–15 coating has a higher concentration of Al element than the MAO–0 and MAO–30 coatings. Figure 3m shows that the MAO–15 coating has the lowest porosity, while the MAO–0 coating exhibits the largest porosity.

**Figure 4:**
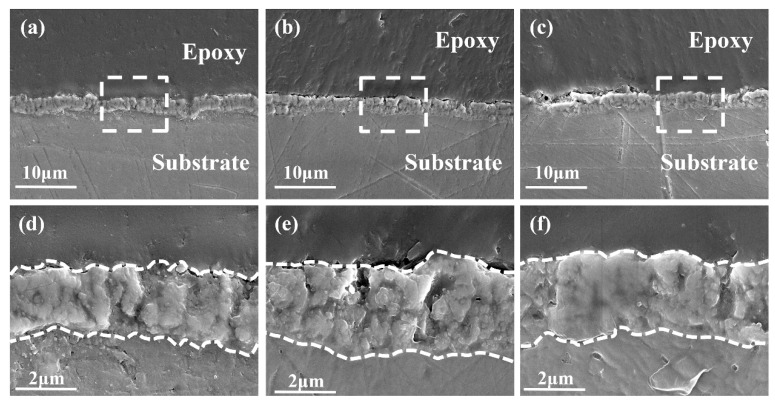
Cross-sectional SEM morphologies of oxide coatings: (**a**,**d**) MAO–0; (**b**,**e**) MAO–15; and (**c**,**f**) MAO–30.

Figure 4 presents the cross-sectional SEM micrographs of the MAO–0, MAO–15, and MAO–30 coatings, demonstrating excellent adhesion between all MAO coatings and the Mg alloy substrate without visible interfacial delamination or cracks [50]. The MAO–0 coating has a thickness of approximately 2.15 ± 0.15 μm, while the MAO–15 shows a slight greater thickness of 3.05 ± 0.65 μm. The MAO–30 displays a thickness of about 2.70 ± 0.20 μm. Higher-magnification images of the framed areas in Figure 4a–c are provided in Figure 4d–f, respectively, revealing a non-uniform coating morphology with numerous voids. These voids correspond to the pores in the coating, indicating that the pores are not only present on the coating surface but also within the coating.

**Figure 5:**
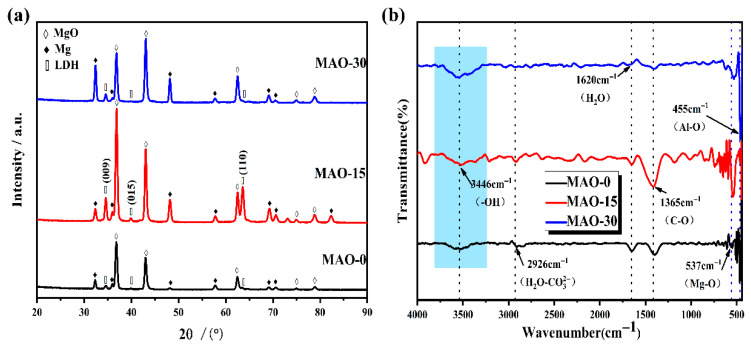
XRD patterns (**a**) and FT-IR spectra (**b**) of MAO–0, MAO–15, and MAO–30 coatings.

The XRD patterns of the MAO-0, MAO–15, and MAO–30 coatings on the AZ91 magnesium alloy, shown in Figure 5a, indicate that the MAO coatings are mainly composed of the MgO phase. Additionally, the diffraction peaks at 34.5°, 39.7°, and 63.4° correspond to the (009), (015), and (110) diffraction planes of the LDH phase, respectively, suggesting that the LDHs were successfully grown on the surface of the MAO coating [51]. Although the MAO–0, MAO–15, and MAO–30 samples do not show significant difference in phase composition, the MAO–15 coating has higher intensity of the diffraction peaks of the LDH phase, which correlates with the differences in surface morphology depicted in Figure 3.

Figure 5b shows the FT-IR spectra of the MAO–0, MAO–15, and MAO–30 coatings. Overall, the spectra exhibit no significant differences. The broad absorption band observed at 3446 cm^−1^ in the FTIR spectrum corresponds to the O-H stretching vibrations originating from both the brucite-like layers of LDH flakes and intercalated water molecules within the interlayer galleries [52]. The absorption peak observed at 2926 cm^−1^ is attributed to the stretching vibration of the H_2_O–${\mathrm{CO}}_{3}^{2-}$ bond [53] and correlates with the intrusion of CO_2_ from the air [37]. The absorption band at 1620 cm^−1^ corresponds to the bending vibration mode δ(H-O-H) of water molecules, characteristic of both physisorbed surface water and interlayer H_2_O in hydrated materials [54]. The 1365 cm^−1^ signal arises from the antisymmetric stretching vibration of C-O, indicative of atmospheric CO_2_ adsorption during sample handling [55]. Other bands within the 800–500 cm^−1^ range are primarily assigned to lattice vibrations involving M-O, M-O-M, and O-M-O bonds [42]. Two bands at 537 cm^−1^ and 455 cm^−1^ are attributed to the Mg-O cohesive group and the Al-O translation, respectively [56,57].

**Figure 6:**
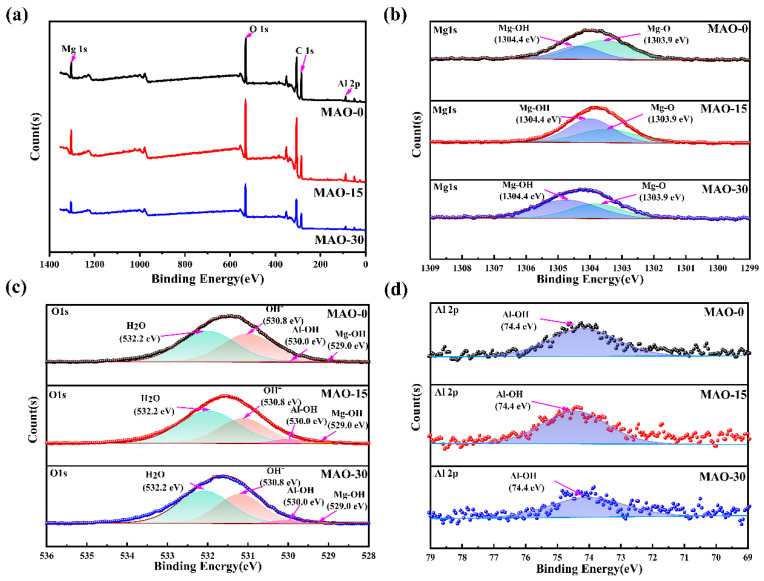
XPS survey spectra of MAO–0, MAO–15, and MAO–30 coatings (**a**) and high-resolution XPS spectra in Mg 1s (**b**), O 1s (**c**), and Al 2p (**d**) spectral regions.

X-ray photoelectron spectroscopy (XPS) was employed to systematically investigate the surface chemical composition and bonding states of the MAO coatings (MAO–0, MAO–15, and MAO–30). As shown in Figure 6b, the Mg 1s peak at 1303.9 eV corresponds to MgO formed during the MAO process. This result confirms that MgO is the primary component of the MAO coating. The Mg 1s peak at 1304.4 eV is attributed to Mg-OH formed on the coating surface [58]. The O 1s spectra at 532.2, 530.8, 530.0, and 529.0 eV have four peaks attributed to H_2_O [42], OH [59], Al-OH [59], and Mg-OH [60], respectively. Al 2p is located at 74.4 eV, which corresponds to Al-OH in the coatings. For the MAO–15 coating, the peaks of Mg-OH in the Mg 1s and the peaks of Al-OH in the O 1s and Al 2p have higher intensity, indicating that the formation of Mg-Al LDHs is more abundant on the surface. This coincides with the surface differences in Figure 3 and also corroborates the XRD and FT-IR (Figure 5) results.

In summary, MAO coatings with Mg-Al LDH flakes on the surface of the magnesium alloy are successfully formed on the AZ91 Mg alloy. The formation mechanism of Mg-Al LDH flakes is proposed as follows. The C_3_H_8_O_3_ added to the electrolyte is a polar molecule that can be easily adsorbed on the Mg alloy due to its strong binding energy with Mg surface [61]. Furthermore, C_3_H_8_O_3_ exhibits high surface activity, which can be attributed to its numerous free radicals and strong polar hydroxyl groups. Therefore, specific adsorption occurs at the interface between Mg alloy substrate and the electrolyte, which subsequently reduces the solid–liquid interfacial tension. The C_3_H_8_O_3_ molecule has the same structure with OH^−^ groups, and it can be inferred that intermolecular repulsive force between the interfacial OH^−^ groups decreases with the specific adsorption of the C_3_H_8_O_3_ molecule. Since they have similar polarity and affinity, OH^−^ groups tend to adsorb with C_3_H_8_O_3_ molecules. Consequently, the adsorption of negative ions at the interface between the Mg alloy surface and the electrolyte is enhanced under alkaline solution conditions. Driven by the electric field during MAO, OH^−^ groups accumulate at the solid–liquid interface, thereby promoting surface reactions on the coating.

The binding energy of oxides within the coating plays a critical role in influencing ion migration in an applied electric field [62]. On the AZ91 Mg alloy substrate, the Gibbs free energy of oxidation per equivalent differs between Mg and the alloying element Al. Thermodynamically, Mg oxidizes preferentially due to its lower Mg-O binding energy (394 kJ/mol) compared to Al-O (512 kJ/mol) during oxidation. As a result, Al^3+^ ions formed on the surface of the coatings migrate outwards more slowly than Mg^2+^ ions, which leads to a gradual enrichment of Al^3+^ ions on the sample surface [45,63]. The degree of enrichment of Al^3+^ ions on the surface is an important factor in the formation of LDHs on the surface of the samples. With the addition of C_3_H_8_O_3_ in the electrolyte, the coating surface reaction during the MAO process is enhanced, with Al participating the oxidation reaction together with Mg, resulting in the formation of the Mg-Al LDHs according to the reactions (1–6). During the microarc oxidation (MAO) process, the addition of C_3_H_8_O_3_ reduces the solid–liquid interfacial tension, promoting the diffusion of the electrolyte at the interface and enhancing the adsorption of negative ions near the anode/electrolyte interface. This, in turn, catalyzes the coating surface reaction. At the initial stage of microarc oxidation, Mg reacts rapidly with H_2_O in the electrolyte to generate MgO under the effect of the applied electric field Meanwhile, the outward migration of Mg^2+^ ions from the Mg alloy substrate is fast, and Mg(OH)_2_ reacts rapidly with OH^−^ under the catalytic effect of C_3_H_8_O_3_ to generate Mg(OH)^3−^ (Equations (1)–(3)). Furthermore, under the effect of the electric field, Al^3+^ ions in the Mg alloy substrate also rapidly form hydroxides in an alkaline condition according to Equations (3) and (4). In alkaline solutions, these reactions with OH^−^ reactions work together to form the Mg-Al LDHs as Equation (5). The main reaction equations are as follows:

Mg + H_2_O → MgO + H_2_↑ (1)

MgO + H_2_O → Mg(OH)_2_ (2)

Mg(OH)_2_ + OH^−^ → Mg(OH)^3−^ (3)

Al^3+^ + 3 OH^−^ → Al(OH)_3_ (4)

Al(OH)_3_ + OH^−^ → Al(OH)_4−_ (5)

(1−x)Mg(OH)^3−^ + xAl(OH)_4−_ + OH^−^ + mH_2_O → [Mg^2+^_1−x_Al^3+^_x_(OH)_2_]^x+^[OH^−^]_x_·mH_2_ (6)

**Figure 7:**
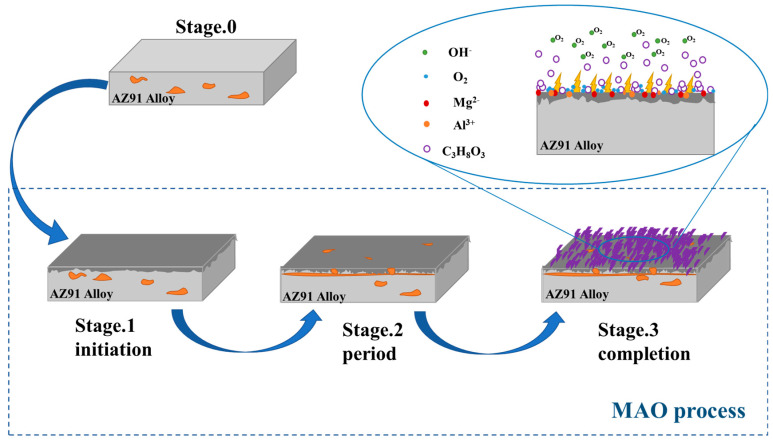
Schematic of the formation process of the MAO coating with Mg-Al LDHs.

Based on the comprehensive characterization results, Figure 7 proposes a mechanistic model for the formation of MAO coatings. During the coating growth process, the surface morphology, as well as the number and size of cracks and pores, varied significantly with different concentrations of C_3_H_8_O_3_. In the absence of C_3_H_8_O_3_, the coating exhibits numerous small-sized pores and cracks. At the same time, a small number of LDH flakes can be observed on the coating surface, which is due to some Mg^2+^ ions and Al^3+^ ions being able to react with the free OH^−^ ions near the solid–liquid interface. With the addition of the C_3_H_8_O_3_, the inter-ion reaction rate is catalyzed, leading to a significant increase in the number of LDH flakes. The coating surfaces then appear tightly packed with continuous LDH flakes. At the same time, the pores and cracks are reduced accordingly, and some of the holes are sealed by the flakes. However, as the C_3_H_8_O_3_ concentration further increases, larger pores form in the coating. This is due to the higher final voltage of the microarc oxidation caused by the increased C_3_H_8_O_3_ content, leading to the appearance of a large, localized arc. Under the effect of high temperature from the large arc, the flakes partially melt, resulting in fewer flakes on the coating surface.

### 3.3. Coating Performance Evaluation

#### 3.3.1. Hydrophobic Effect

**Figure 8:**
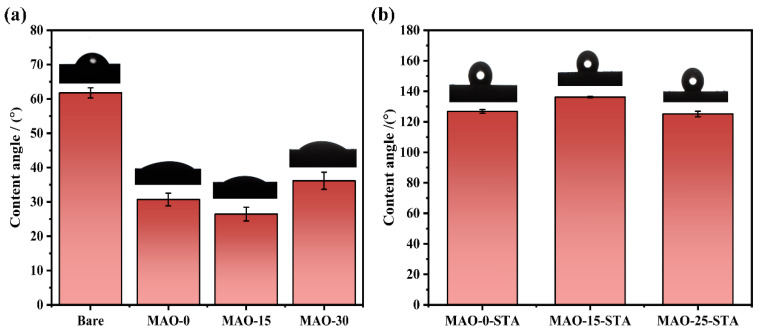
Water contact angle of AZ91 Mg alloy substrate, MAO–0, MAO–15, and MAO–30 coatings (**a**) and MAO–0–STA, MAO–15–STA, and MAO–30–STA coatings (**b**).

The surface wettability of the bare Mg alloy substrate, MAO–0, MAO–15, and MAO–30 coatings was characterized. Figure 8a shows that the water contact angle (WCA) of the bare Mg alloy substrate is approximately 61.76°. However, the WCAs for MAO–0, MAO–15, and MAO–30 are significantly reduced to 30.70°, 26.50°, and 36.15°, respectively, indicating that the MAO coatings exhibit good hydrophilicity. The hydrophilicity of the MAO coatings can be ascribed to the chemical compositions and surface microstructure. MgO is a compound with very good hydrophilicity. Furthermore, the roughness of the surface along with the pores and cracks make it more hydrophilic, especially for the MAO–15 coating. This observation is consistent with Wenzel’s theory, which suggests that increasing the surface roughness of a hydrophilic material enhances its wettability [64]. However, surface modification by using stearic acid (STA) significantly increases the water contact angles (WCAs) of the MAO coatings. As can be seen from Figure 8b, the MAO–0–STA, MAO–15–STA, and MAO–30–STA coatings show WCAs of 126.85°, 136.17°, and 125.12°, respectively. Notably, MAO–15–STA exhibits the largest water contact angle, deviating from hydrophilic property before modification to a near-superhydrophobic effect after modification. This phenomenon is primarily attributed to both the modification of STA and the distinctive hierarchical micro-nanostructures of LDHs formed on the coating surface.

**Figure 9:**
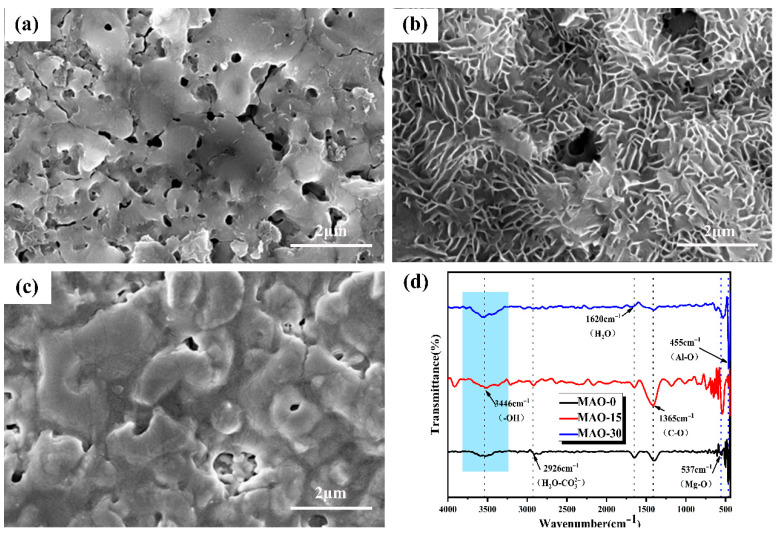
Surface morphologies (**a**–**c**) and FT-IR spectra (**d**) of MAO–0–STA, MAO–15–STA, and MAO–30–STA coatings.

Figure 9 shows the surface morphology and FT-IR spectra of the MAO coatings after surface modification. It can be seen from Figure 9a–c that the nano-lamellar structures of the MAO–0 and MAO–30 coatings nearly disappear, which is mainly due to the high temperature. For the MAO-15 coating, however, the lamellar structure is maintained after surface modification. The FTIR spectra (Figure 9d) reveal characteristic absorption bands at 1545 cm^−1^ and 1455 cm^−1^, which are assigned to the stretching vibrations of carbonyl (C=O) functional groups. These signatures indicate asymmetric C=O stretching in carboxylate moieties and symmetric C=O stretching or possible carbonate contamination [65], indicating the successful grafting of STA on the surface of the coatings.

#### 3.3.2. Corrosion Resistance

**Figure 10:**
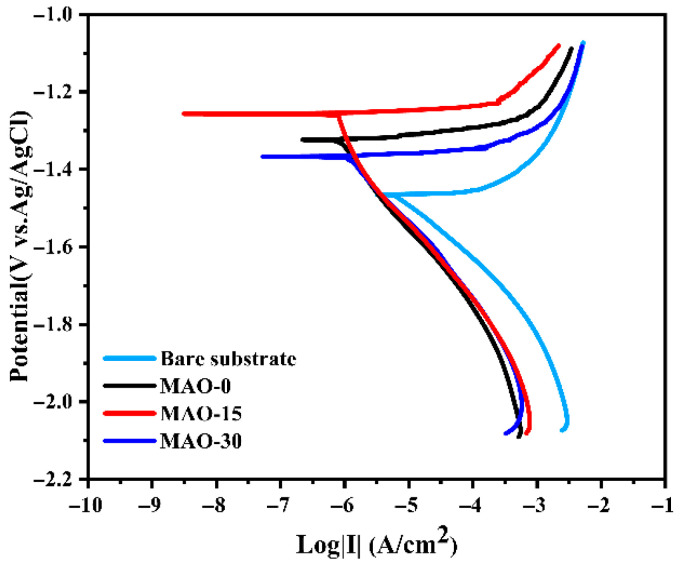
Potentiodynamic polarization curves of bare and MAO-coated AZ91 Mg alloy in 3.5 wt.% NaCl solution.

The corrosion resistance of bare and microarc oxidation (MAO)-coated AZ91 Mg alloys was systematically evaluated using electrochemical techniques complemented by hydrogen evolution measurements. Potentiodynamic polarization tests in 3.5 wt.% NaCl solution (Figure 10) revealed clear differences in electrochemical behavior between uncoated and MAO-treated samples. Through Tafel extrapolation analysis, critical parameters, including corrosion potential (*E*_corr_) and current density (*i*_corr_), were quantitatively determined (Table 1). Notably, the *E*_corr_ values demonstrated a significant positive shift for MAO-coated specimens (−1.332 V for MAO–0, −1.259 V for MAO–15, and −1.367 V for MAO-30) compared to the bare alloy (−1.473 V), indicating enhanced corrosion protection. From kinetic and thermodynamic perspectives, the corrosion resistance of the coatings exhibits a positive correlation with their corrosion potential (*E*_corr_). The MAO–15 coating demonstrates the highest *E*_corr_ value among all tested samples (−1.259 V vs. reference electrode), indicating its superior corrosion resistance in the aggressive 3.5 wt.% NaCl environment. For the MAO-coated samples, the MAO–15 coating exhibits the lowest corrosion current density (*i*_corr_ = 4.88 × 10^−7^ A/cm^2^), indicating its superior corrosion protection. This improvement can be attributed to the sealing effect of LDHs on the pores and cracks within the coating. Furthermore, the MAO-30 coating shows reduced corrosion resistance compared to the MAO–15 coating. This can be ascribed to the formation of larger pores on the coating surface caused by strong arcs during the MAO process (Figure 2b and Figure 3a–i), which decreases the integrity of the coating.

**Figure 11:**
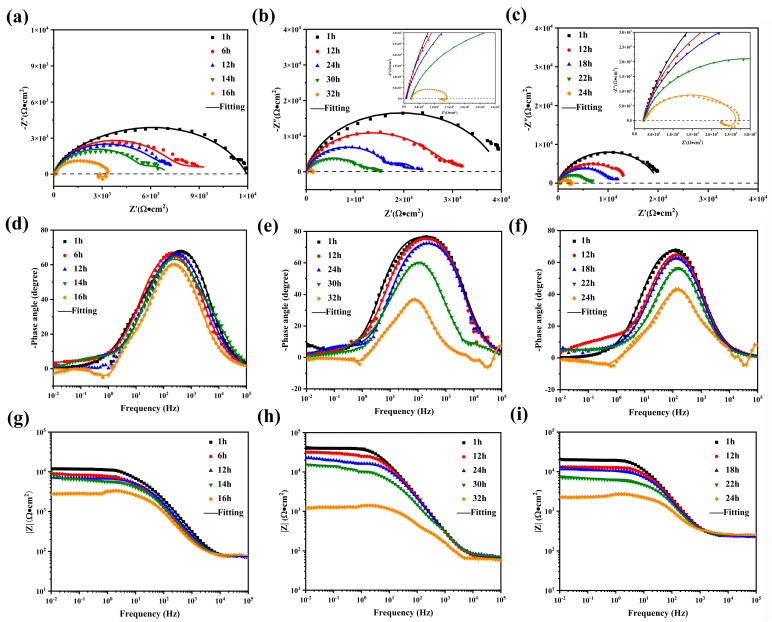
Electrochemical impedance spectra of MAO-coated Mg alloys in 3.5 wt% NaCl solution: (**a**,**d**,**g**) MAO–0 coating, (**b**,**e**,**h**) MAO–15 coating, (**c**,**f**,**i**) MAO–30 coating.

Electrochemical impedance spectroscopy (EIS) was systematically employed to evaluate the corrosion protection performance of the MAO coatings. The Nyquist and Bode plots are presented in Figure 11. In general, the capacitive loop diameter can be indicative of the overall corrosion resistance of the coating. A larger axial radius of the capacitive loop signifies better corrosion resistance, whereas the appearance of the inductive loop is attributed to the corrosion behavior of the magnesium substrate [19]. From the Nyquist plots (Figure 11a–c), it can be seen that the MAO–15 coating exhibits the largest radius of the capacitive loop, while the MAO–0 coating showed the smallest radius of capacitive loop after 1 h immersion in 3.5 wt% NaCl solution, indicating that the MAO–15 coating has much better corrosion resistance than the MAO–0 coating. The MAO–0 and MAO–30 coatings show inductive loops after 16h and 24h immersion tests, respectively, which means that the corrosive medium has penetrated through the coating to corrode the Mg alloy substrate. Furthermore, by analyzing the |Z|_0.01Hz_ values from Bode plots, it is possible to assess and compare the corrosion resistance among various specimens. As observed, the|Z|_0.01Hz_ values of all coatings decreased with the immersion time, suggesting a gradual decrease in the corrosion protection of the coatings. As shown in Figure 11d,f, the |Z|_0.01Hz_ values of the MAO–0 and MAO–30 coatings decrease significantly to less than 10^4^ Ω⋅cm^2^ after longer immersion. The phase–frequency Bode plots (Figure 11g,i) reveal negative phase angles (<0°) at low frequencies (0.01–0.1 Hz) for MAO coatings, further indicating a significant reduction in the protective performance of the MAO coating. When comparing the overall |Z|_0.01Hz_ values, the corrosion resistance ranking is MAO–15 > MAO–30 > MAO–0, further confirming the enhanced corrosion resistance provided by the LDHs on the MAO–15 coating.

**Figure 12:**
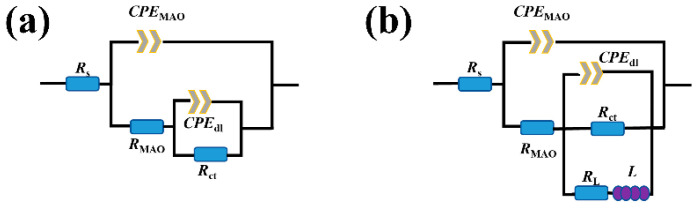
Equivalent circuits used to fit EIS data: (**a**) the EIS data of the MAO–0 coating after 1, 6, 12, and 14 h of immersion; MAO–15 coating after 1, 12, 24, and 30 h of immersion;and MAO–30 coating after 1, 12, 18, and 22 h; (**b**) the EIS data of the MAO–0 coating after 16 h of immersion and MAO–15 coating after 32 h. [45].

The EIS plots in Figure 11 were fitted to further analyze the corrosion behavior of the coatings. According to the EIS plots, two equivalent circuit (EC) models were proposed to fit the EIS data, respectively. The EC models depicted in Figure 12a were utilized for the EIS data of the MAO–0 coating after 1, 6, 12, and 14 h of immersion; MAO–15 coating after 1, 12, 24, and 30 h of immersion; and MAO–30 coating after 1, 12, 18, and 22 h of immersion, while the EIS data of the MAO–0 coating after 16 h of immersion and MAO–15 coating after 32 h after immersion are consistent with the EC model shown in Figure 12b. [66]. *R*_s_ represents the solution resistance, while *CPE*_MAO_ and *CPE*_dl_ correspond to the capacitance of the MAO coating and the capacitance of the electrical double layer at the coating/substrate interface, respectively, and *R*_MAO_ and *R*_ct_ correspond to the resistance of the MAO coating and charge transfer resistance at the interface, respectively. In addition, an inductive element (*L*) and inductance resistance (*R*_L_) are used to represent the inductance behavior of the MAO coating. The corresponding fitting results are listed in Table 2, Table 3 and Table 4.

**Figure 13:**
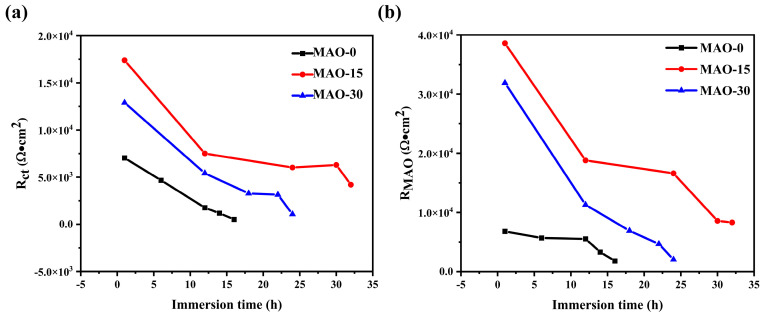
The evolution of (**a**) *R*_MAO_ and (**b**) *R*_ct_ values of MAO–0, MAO–15, and MAO–30 coatings as a function of immersion time.

The overall corrosion resistance of the coatings, which is indicated by the changes in coating resistance (*R*_MAO_) and charge transfer resistance (*R*_ct_) over time of immersion, is shown in Figure 13. A higher *R*_MAO_ value reflects better durability and protective performance. Charge transfer resistance (*R*_ct_) reflects the kinetic barrier for ion migration across the double layer, with higher values indicating superior corrosion resistance. As shown in Figure 13, the MAO–0 coating has the lowest *R*_MAO_ value, which is only 1.80 × 10^3^ Ω/cm^2^ after immersion for 16 h. The MAO–15 coating still has a high *R*_MAO_ value after immersion for 30 h, indicating that this coating has better durability and protection performance. It is worth noting that the *R*_MAO_ value of the MAO–15 coating also decreased significantly after a long time of immersion, which may be related to the fact that the coating has a hydrophilic effect and is easily penetrated by corrosive medium [44]. Furthermore, the *R*_ct_ values also exhibit a similar trend, highlighting that the MAO–15 coating possesses the highest *R*_ct_ value. In summary, the corrosion protection performance of the coatings evaluated by EIS tests is consistent with the results of potentiodynamic polarization curves.

**Figure 14:**
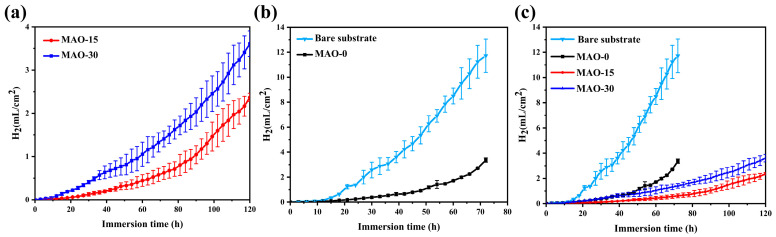
Cumulative hydrogen volume (specific volume: volume per unit area of exposed surface) vs. immersion time in 3.5 wt.% NaCl solution: (**a**) cumulative hydrogen volume; (**b**) comparison of cumulative hydrogen volume between bare pure AZ91 alloy and MAO–0; (**c**) comparison of cumulative hydrogen volume between MAO–15 and MAO–30.

It is known that the hydrogen evolution measurements can be used to indicate the dissolution of the Mg substrate and the corrosion protection performance of the coatings. The cumulative hydrogen volume versus immersion time of bare AZ91 Mg alloy and MAO coatings immersed in 3.5 wt.% NaCl solution is shown in Figure 14. It can be seen from Figure 14a,b that the bare AZ91 Mg alloy has a much higher hydrogen evolution rate than the MAO coatings. In contrast, the MAO–0 coating shows a significant increase in the hydrogen evolution rate after 50 h of immersion, which can be attributed to the corrosion of the Mg alloy. In comparison, the MAO–15 coating demonstrates the least hydrogen evolution throughout the immersion process, suggesting the best corrosion protection. The experimental results are in good agreement with the electrochemical corrosion tests.

Both electrochemical analyses (Figure 10 and Figure 11) and hydrogen evolution tests (Figure 14) indicate that the MAO–15 coating with large number of LDHs exhibit superior corrosion resistance. On one hand, the LDHs partially seal the defects of the MAO coating, thereby enhancing its physical barrier properties. On the other hand, LDHs are able to trap Cl^−^ in the corrosive medium and form an ion-enriched barrier on the surface due to the greater exchange of OH^−^ anions than Cl^−^ [34,35,67]. However, it can be observed that the corrosion resistance of the MAO–15 coating also showed a significant decrease at the late stage of immersion, which is mainly attributed to the fact that the defects of the MAO–15 coating were not completely sealed by the LDHs (Figure 3). In addition, the MAO–15 coating exhibits a noticeable hydrophilic effect, which is beneficial for the absorption of more corrosive medium to deteriorate the long-term protection performance [68].

**Figure 15:**
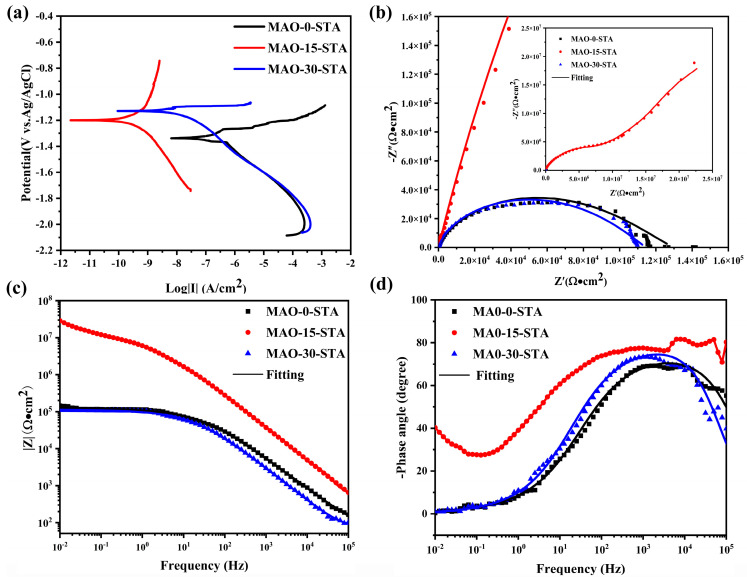
Electrochemical corrosion testing results of MAO–0–STA, MAO–15–STA, and MAO–30–STA coatings in 3.5 wt% NaCl solution: (**a**) potentiodynamic polarization curves, (**b**) EIS Nyquist plots, (**c**) EIS impedance–frequency Bode plots, and (**d**) EIS phase–frequency Bode plots.

The corrosion properties of the MAO coatings modified by stearic acid (STA) were also evaluated. Figure 15a shows the potentiodynamic polarization curves of the MAO–0–STA, MAO–15–STA, and MAO–30–STA coatings in 3.5 wt% NaCl solution, and the fitting results are shown in Table 5. Compared with the MAO–0–STA film, the MAO–15–STA and MAO–30–STA films exhibit a positive shift in corrosion potential and reduced corrosion current density, indicating that the STA modification greatly decreases the corrosion tendency of the coatings. The electrochemical impedance spectra of the STA modified coatings in 3.5 wt% NaCl solution are presented in Figure 15b–d. The corresponding fitting results are listed in Table 6, and the equivalent circuit diagrams used for the fitting are shown in Figure 12a and Figure 15b. Warburg impedance is introduced in Figure 15b, and (*Z*_w_) is the diffusion resistance, which embodies the diffusion ability of the coating, and is used to characterize the interlayer ion exchange process of the LDHs [35]. The larger (*Z*_w_) of the coating indicates that it has a better ion exchange ability, which is conducive to minimizing the presence of Cl^−^ on the surface of the film in the corrosive solution, thus slowing down the corrosion process. From Table 6, it can be seen that the MAO–STA coatings have greatly higher *R*_MAO_ values when compared to the MAO coatings. This is mainly attributed to the hydrophobicity of the MAO–STA coatings enhancing the protective performance. Among them, MAO–15–STA exhibits a much lower corrosion current density compared to the MAO-0-STA and MAO–30–STA coatings. Furthermore, the MAO–15–STA coating has the largest *R*_ct_ values and *R*_MAO_ values according to Table 6, which shows the best corrosion protection performance.

It is evident that the improved corrosion protection of the coatings can be attributed to the hydrophobicity resulting from modification with stearic acid. The hydrophobic properties reduce the contact area between the sample surface and the corrosive medium, thereby decreasing the penetration of corrosive medium. The formation of well-defined LDHs on the MAO–15 coating further enhances the hydrophobic effect, which contributes to the best corrosion resistance by acting as a barrier against the corrosive medium. The hydrophobic organic layer introduced by stearic acid modification effectively reduces the direct contact area between corrosive media and the coating through a physical barrier effect, thereby significantly enhancing the protective performance of the coating. This hydrophobic effect, in conjunction with the ion exchange and physical sealing functions of the LDH layer, collectively contributes to the excellent corrosion resistance of the composite coating.

## 4. Conclusions

In this study, MAO coatings with Mg–Al–LDHs were fabricated on AZ91 Mg alloy in a low-voltage process. The main findings are as follows:

1. Mg-Al LDHs with hydroxides were formed in situ on Mg alloy under low voltage conditions with a high concentration of NaOH electrolyte and the addition of C_3_H_8_O_3_.
2. The necessary species for the formation of the Mg-Al LDHs include Mg^2+^, Al^3+^, and OH^−^. Mg^2+^ and Al^3+^ are derived from the AZ91 Mg alloy substrate, while OH^−^ is provided by the alkaline electrolyte. Introducing C_3_H_8_O_3_ into the electrolyte enhances the surface reaction and facilitates the formation of Mg-Al LDHs. The optimal concentration of C_3_H_8_O_3_ is 15 mL/L.
3. The MAO coating with Mg–Al–LDHs on AZ91 magnesium alloy originally exhibited hydrophilicity and transformed into a nearly superhydrophobic state after surface modification by stearic acid.
4. The MAO coating with Mg–Al–LDHs on AZ91 magnesium alloy showed enhanced corrosion resistance. After surface modification, the corrosion current density (*i*_corr_) decreased by 6 orders of magnitude, indicating a significant improvement in corrosion protection.

## Figures and Tables

**Table 1:** Corrosion current densities (*i*_corr_) and corrosion potential (*E*_corr_) derived from the polarization measurements of the samples.

Material	*i*_corr_ (A/cm^2^)	*E*_corr_ (V)
Bare	1.05 × 10^−4^	−1.473
MAO–0	1.03 × 10^−6^	−1.332
MAO–15	4.88 × 10^−7^	−1.259
MAO–30	1.63 × 10^−6^	−1.367

**Table 2:** Fitting results of EIS plots of MAO–0 coating.

Immersion Time (h)	*R*_s_ (Ω/cm^2^)	*CPE*_MAO_ (F/cm^2^)	*CPE*_MAO_-n	*R_M_*_AO_ (Ω/cm^2^)	*CPE*_dl_ (F/cm^2^)	*CPE*_dl_-n	*R*_ct_ (Ω/cm^2^)	*R*_L_ (Ω/cm^2^)	*L* (H/cm^2^)
1	73.59	3.27 × 10^−7^	0.90	6.8 × 10^3^	8.58 × 10^−6^	0.70	7.04 × 10^3^	-	-
12	73.63	2.25 × 10^−7^	0.85	5.69 × 10^3^	7.83 × 10^−6^	0.67	4.67 × 10^3^	-	-
24	73.6	2.17 × 10^−7^	0.87	5.53 × 10^3^	4.92 × 10^−6^	0.63	1.76 × 10^3^	-	-
30	74.16	3.27 × 10^−6^	0.84	3.30 × 10^3^	3.86 × 10^−6^	0.58	1.18 × 10^3^	-	-
32	76.69	9.67 × 10^−5^	0.83	1.80 × 10^3^	6.96 × 10^−7^	0.72	5.19 × 10^2^	3098	3567

**Table 3:** Fitting results of EIS plots of MAO–15 coating.

Immersion Time (h)	*R*_s_ (Ω/cm^2^)	*CPE*_MAO_ (F/cm^2^)	*CPE*_MAO_-n	*R*_MAO_ (Ω/cm^2^)	*CPE*_dl_ (F/cm^2^)	*CPE*_dl_-n	*R*_ct_ (Ω/cm^2^)	*R*_L_ (Ω/cm^2^)	*L* (H/cm^2^)
1	67.45	1.32 × 10^−6^	0.90	3.86 × 10^4^	9.49 × 10^−6^	0.81	1.74 × 10^4^	-	-
12	71.53	1.17 × 10^−6^	0.92	1.88 × 10^4^	7.61 × 10^−6^	0.77	7.51 × 10^3^	-	-
24	71.25	1.56 × 10^−6^	0.88	1.66 × 10^4^	6.47 × 10^−6^	0.76	6.02 × 10^3^	-	-
30	234.8	2.79 × 10^−6^	0.86	8.58 × 10^3^	2.33 × 10^−6^	0.55	6.30 × 10^3^	-	-
32	243.6	6.64 × 10^−6^	0.95	8.31 × 10^3^	1.14 × 10^−7^	0.78	4.20 × 10^3^	1.41 × 10^3^	9547

**Table 4:** Fitting results of EIS plots of MAO–30 coating.

Immersion Time (h)	*R*_s_ (Ω/cm^2^)	*CPE*_MAO_ (F/cm^2^)	*CPE*_MAO_-n	*R*_MAO_ (Ω/cm^2^)	CPE_dl_ (F/cm^2^)	*CPE*_dl_-n	*R*_ct_ (Ω/cm^2^)	*R*_L_ (Ω/cm^2^)	*L* (H/cm^2^)
1	232	1.41 × 10^−6^	0.89	3.19 × 10^4^	5.49 × 10^−6^	0.73	1.29 × 10^4^	-	-
12	235.7	1.51 × 10^−6^	0.90	1.13 × 10^4^	2.37 × 10^−6^	0.69	5.42 × 10^3^	-	-
18	232.9	2.05 × 10^−6^	0.88	6.91 × 10^3^	1.88 × 10^−6^	0.64	3.28 × 10^3^	-	-
22	245.7	3.27 × 10^−6^	0.89	4.67 × 10^3^	1.29 × 10^−6^	0.59	3.14 × 10^3^	-	-
24	76.69	7.17 × 10^−6^	0.76	2.04 × 10^3^	3.77 × 10^−7^	0.76	1.07 × 10^3^	3887	4829

**Table 5:** Corrosion potential (*E*_corr_) and corrosion current densities (*i*_corr_) after surface modification of the specimen.

Material	*i*_corr_ (A/cm^2^)	*E*_corr_ (V)
MAO-0-STA	2.78 × 10^−7^	−1.329
MAO-15-STA	2.86 × 10^−10^	−1.193
MAO-30-STA	4.12 × 10^−9^	−1.124

**Table 6:** Fitting results of MAO-0-STA, MAO-15-STA, and MAO-30-STA coatings immersed in 3.5 wt% NaCl solution for 30 min.

Samples	*R*_s_ (Ω/cm^2^)	*CPE*_MAO_ (F/cm^2^)	*CPE*_MAO_-n	*R*_MAO_ (Ω/cm^2^)	*CPE*_dl_ (F/cm^2^)	*CPE*_dl_-n	*R*_ct_ (Ω/cm^2^)	*Z*_w_ (Ω^−0.5^·S^−1^·cm^−2^)
MAO–0–STA	68.89	1.03 × 10^−7^	0.84	7.56 × 10^6^	7.81 × 10^−7^	0.39	1.29 × 10^5^	-
MAO–15–STA	43.82	2.77 × 10^−8^	0.87	2.25 × 10^7^	2.14 × 10^−8^	0.89	7.09 × 10^6^	1.71 × 10^−7^
MAO–30–STA	68.06	7.82 × 10^−7^	0.48	1.05 × 10^7^	1.05 × 10^−7^	0.91	1.14 × 10^5^	-

## Data Availability

The original contributions presented in this study are included in the article. Further inquiries can be directed to the corresponding authors.
